# Serological Survey of Lyssaviruses in Polish Bats in the Frame of Passive Rabies Surveillance Using an Enzyme-Linked Immunosorbent Assay

**DOI:** 10.3390/v12030271

**Published:** 2020-02-28

**Authors:** Anna Orłowska, Marcin Smreczak, Conrad Martin Freuling, Thomas Müller, Paweł Trębas, Jerzy Rola

**Affiliations:** 1 Department of Virology, National Veterinary Research Institute, 24-100 Puławy, Poland; pawel.trebas@piwet.pulawy.pl (P.T.); Jerzy.Rola@piwet.pulawy.pl (J.R.); 2 Institute of Molecular Virology and Cell Biology, FLI, WHO Collaborating Centre for Rabies Surveillance and Research, OIE Reference Laboratory for Rabies, 17493 Greifswald-Insel Riems, Germany; Conrad.Freuling@fli.de (C.M.F.); Thomas.Mueller@fli.de (T.M.)

**Keywords:** rabies, lyssaviruses, seroprevalence, bats, Poland, ELISA

## Abstract

*Background*: Bats are known to host a number of nonpathogenic viruses, as well as highly pathogenic viruses causing fatal diseases like rabies. Serological surveys as part of active and passive bat rabies surveillance mainly use seroneutralization assays, demonstrating the presence of lyssavirus-specific antibodies in a variety of European bats, particularly against European bat lyssaviruses type 1 (EBLV-1). Here, we present the first serological survey in European bats of this kind during which European bats from Poland collected in the frame of passive rabies surveillance between 2012 and 2018, as well as Serotine bats (*Eptesicus serotinus*) and North American Big Brown bats (*Eptesicus fuscus*) from previous experimental studies, were tested using a commercial ELISA kit for the detection of anti-lyssavirus antibodies. *Results*: Lyssavirus-specific antibodies were detected in 35 (30.4%) out of 115 Polish bats of both sexes, representing nine out of 13 identified bat species endemic mainly to Central Southern Europe and Western Asia, i.e., *Eptesicus serotinus*, *Nyctalus noctula*, *Myotis daubentonii*, *Plecotus auritus*, *Vespertillo murinus,Pipistrellus pipistrellus*, *Pipistrellus pipilstrellus/Pipistrellus pygmaeus*, *Myotis brandtii*, and *Barbastella barbastellus*. Seroprevalence was highest in bat species of *Nyctalus noctula, Eptesicus serotinus, Plecotus auritus*, and *Myotis daubentonii*. More than 60% of the ELISA seropositive bats originated from the voivodeships of Silesia, Lower-Silesian, Warmian-Mazurian, and Mazowian. Rabies-specific antibodies were also found in *Eptesicus fuscus* bats from North America. *Conclusions*: The study demonstrates the principal application of the BioPro Rabies ELISA Ab Kit for the detection of anti-lyssavirus specific antibodies in body fluids and serum samples of bats. However, results may only be reliable for North American bats, whereas interpretation of results for European bats per se is difficult because proper validation of the test is hampered by the protected status of these species.

## 1. Introduction

Bats (*Chiroptera*) are reservoirs of a large number of viruses, some of which can cause infections and diseases in animals and humans [1]. One example is rabies, an acute viral infection of the central nervous system caused by viruses belonging to the Mononegavirales order, Rhabdoviridae family, and Lyssavirus genus [2]. Based on demarcation criteria including antigenic and genetic distances, 16 lyssavirus species are accepted by the International Committee on the Taxonomy of Viruses [3]. Lyssaviruses are evolutionarily linked with Chiroptera, and all except for Mokola and Ikoma lyssaviruses were isolated from different species of bats [4]. Moreover, the recently discovered viruses Kotalahti bat lyssavirus (KBLV) and Taiwan bat lyssavirus (TWBLV) were also isolated in Myotis (*Myotis brandtii*) and Pipistrelle (*Pipistrellus abramus*) bats, respectively [5,6].

While, in the Americas, rabies in bats is exclusively caused by the prototypical rabies virus (RABV), for reasons unknown, bats in other parts of the world seem to harbor a diversity of lyssaviruses [4]. In Europe, for example, six different species of lyssaviruses are known to circulate in insectivorous bats. Here, since the first reported case of bat rabies from Germany in 1954, more than 1200 cases of bat rabies were reported, which were mainly detected in Central Europe ranging from the north European Plain to the British Isles and the Iberian Peninsula. While the vast majority of bat rabies cases in Europe are caused by European bat lyssavirus 1 (EBLV-1) infection, mainly associated with Serotine bats (*Eptesicus serotinus, Eptesicus isabellinus*) [4], the remaining five lyssavirus species are only sporadically detected. Two dozen cases of European bat lyssavirus 2 (EBLV-2) were found in Daubentons bats (*Myotis daubentonii*) and Pond bats (*Myotis dasycneme*) from the United Kingdom (UK), the Netherlands, Germany, Finland, Norway, and Switzerland [7]. Other lyssaviruses isolated from European bats are Bokeloh bat lyssavirus (BBLV) in *Myotis nattereri* from Germany, France, and Poland [8,9], Lleida bat lyssavirus (LLEBV) in *Miniopterus schreibersii* from Spain and France [10,11,12], and the recently discovered Kotalahti bat lyssavirus (KBLV) from Finland [5]. Moreover, a single case of West Caucasian bat virus was detected in *Miniopterus schreibersii* from Russia [13]. 

In Poland, the first case of bat rabies was reported in 1972 in a bat of the Vespertillionidae family, presumably a Serotine bat, followed by further sporadic detection of cases in 1985, 1990, and 1995 [14]. Since 1998, continuous passive bat rabies surveillance resulted in an increasing number of bats submitted for diagnosis. By the year 2018, a total of 119 cases of EBLV-1 and one case of BBLV were detected in *Eptesicus serotinus* [15] and *Myotis nattereri*, respectively [8].

In Europe, all bats are legally protected; thus, any surveillance activity is subject to regulations. Although both active and passive rabies surveillance schemes are used across Europe, surveillance varies considerably from country to country in terms of intensity and frequency [16]. Passive surveillance is the method of choice to analyze the occurrence of rabies in bats. This approach comprises post-mortem investigations of diseased bats or bats found dead for lyssavirus antigen in brain samples. In contrast, active surveillance studies focus on the investigation of the presence of virus or viral RNA in oro-pharyngeal swab samples or lyssavirus-specific antibodies in serum samples from free-living bat populations [17]. Such studies are inherently complex, as the catching of bats, sampling, and subsequent diagnostic analyses need to be well coordinated. Because lyssaviruses are excreted only intermittently [2] and, for EBLV-1 and 2, likely at a level below the limit of detection as experimentally shown [18,19,20], the probability of detecting viable virus or viral RNA in bat saliva during active surveillance field studies is very low [21,22]. For serological studies, modified versions of labor-intensive serum neutralization tests (SNTs) are, thus, exclusively used [21,23,24,25,26,27,28,29,30]. Particularly for bat sera, these tests represent a challenge in terms of interpretation of test results, as (i) it is often difficult to distinguish specific from unspecific reactions due to a lack in standardization, and (ii) the limited amount of serum samples restricts serological testing to only one of the six circulating lyssaviruses, mostly EBLV-1, even in non-reservoir species [16,21].

Enzyme-linked immunosorbent assays (ELISA) may circumvent inherent limitations concerning the number of tested samples and the interpretation of results observed with SNTs. ELISA tests for detection of rabies-specific antibodies in dogs and wildlife were developed [31,32,33]. The commercially available BioPro Rabies Ab ELISA kit as a competition ELISA allows for species-independent detection of rabies-specific binding antibodies, and it was successfully used in the frame of monitoring of oral rabies vaccination campaigns [34]. However, a recent amendment of the manufacturer’s instructions offers the possibility to also test bat sera for the presence of anti-lyssavirus antibodies in bat serum samples and body fluids. In this study, we tested the utility of the commercial BioPro Rabies ELISA Ab kit for future serosurveys in the frame of active surveillance using a panel of European and North American bat sera. 

## 2. Materials and Methods 

### 2.1. Samples

In this study, 115 body fluids from Polish bats found dead and collected in the frame of passive rabies surveillance were included. The sampling was performed between 2012 and 2018 in different voivodships of Poland. Except for two bats, which tested positive by the Fluorescent Antibody Test (FAT) for detection of lyssavirus antigen [35], all other bats tested negative when examined by Polish regional veterinary laboratories. Bat carcasses were then submitted to the National Veterinary Research Institute (NVRI) for further research including molecular identification of bat species and detection of anti-lyssavirus antibodies. Some of the bat carcasses were kept frozen for several years before testing. After thawing of bat carcasses, body fluids were sampled during necropsy, i.e., upon availability, blood was sampled from the heart chambers and body fluids were taken from the thoracic cavity. Subsequently, serum was obtained after centrifugation of blood at 2500 rpm, at 4 °C for 10 min. All samples were kept frozen at −20 °C until ELISA examination.

For the two bats that tested positive in FAT, EBLV-1 was detected using a heminested RT-PCR method as described elsewhere [36]. Next, lyssavirus typing was performed based on the nucleotide sequence analysis of N gene fragment and multiple sequence alignment to the reference isolates. For each bat specimen, the sex was estimated, and body fluids or serum samples were taken from all bat individuals during bat necropsy. 

Additionally, 87 bat sera were obtained from previous experimental studies in *Eptesicus serotinus (n =* 27) [18] and North American big brown bats (*Eptesicus fuscus, n* = 60) [19]. All blood samples were taken from the vena uropatagialis prior to infection, thus resembling active surveillance samples. Of note, for *Eptesicus fuscus*, animals were bled twice within an interval of 105 days. 

### 2.2. Molecular Classification of Polish Bats Based on Genetic Markers

Molecular identification of bat species was performed based on cytochrome b sequence analyses as described before [37]. Briefly, around 25-mm^2^ fragments of the wing membrane were sampled from all bat specimens. Total DNA was isolated from wing samples using DNA Mini Kit (Qiagen) after overnight lysis using proteinase K. The lysate was loaded on DNA spin columns following the manufacturer’s instructions, and the resulting DNA was used for amplification of the cytochrome b fragment as published before [37]. Amplicons were subjected to Sanger sequencing in both directions on the automated sequencer ABI PRISM 310 Genetic Analyzer (Applied Biosystem/Thermo Fisher Scientific, Waltham, MA, USA) using a BigDye Sequencing Kit (Applied Biosystem/Thermo Fisher Scientific, Waltham, MA, USA) with GeneScan Analysis Software and the same primers as used for PCR. Bat classification was performed based on sequence mapping with reference sequences using the Basic Local Alignment Search Tool (BLAST) algorithm. 

### 2.3. Detection of Lyssavirus Antibodies with Bio-Pro Rabies ELISA Ab Kit

Antibodies against lyssaviruses were detected using the commercially available BioPro Rabies ELISA Ab kit (O.K. SERVIS BioPro, Prague, Czech Republic), a blocking ELISA for the serological detection of rabies antibodies in domesticated [38] and wild animals [39]. In brief, following manufacturer’s instruction on the modification of the ELISA for bat sera, 10 µL of bat sample was diluted 10-fold to a total of 100 µL using the sample diluent buffer provided in the kit. Additionally, the kit’s controls were also diluted two-fold, and all samples were distributed into the respective wells of the plate. 

Thereafter, the plate was sealed and incubated at 4 °C for 24 h with gently stirring. The subsequent workflow was performed according to the manufacturer’s instructions, essentially as described before [40]. 

The percentage of blocking (PB) or inhibition was calculated for each sample according to the manufacturer’s recommendations (i.e., PB = [(ODNC − ODsample)/(ODNC − ODPC)] × 100, where ODNC is the optical density of the negative control, ODPC is the optical density of the positive control, and ODsample is the optical density of the sample. For bat sera and body fluids, a cut-off of PB ≥ 30% was considered positive for lyssavirus antibodies according to the manufacturer’s instructions.

### 2.4. Modified Rapid Fluorescent Foci Inhibition Test (RFFIT)

The presence of virus neutralizing antibodies against EBLV-1 in bat sera was determined with a modified RFFIT using EBLV-1 as a challenge virus [41]. Sera were tested in two-fold dilutions on mouse neuroblastoma cells (MNA 42/13) with a starting dilution of 1:10.

## 3. Results

### 3.1. Serological Survey of Polish Bats

**Figure 1:**
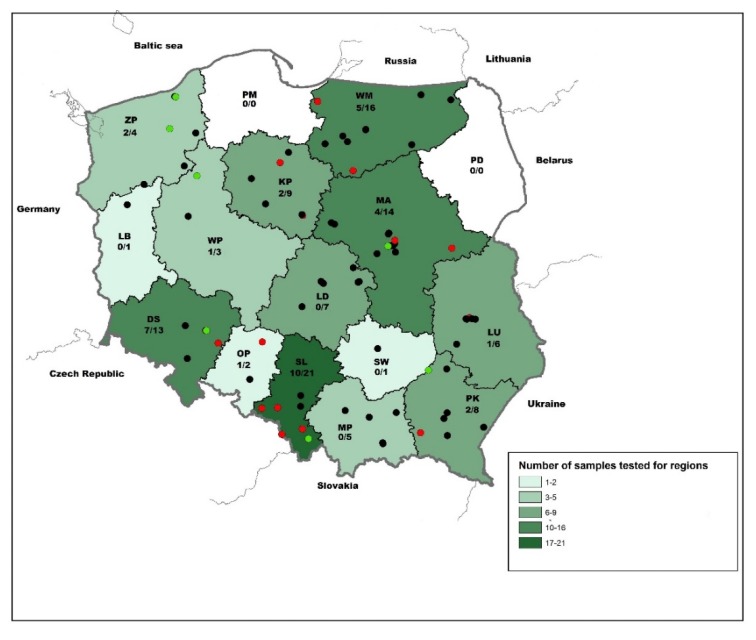
Map showing the voivodeships of Poland and the origin of bats included in the study and ELISA results (black: negative, green: percentage of blocking (PB) > 30%; red: PB > 70%). The number of positive to total samples per region is indicated. Abbreviations: MA (Masovia), DS (Lower-Silesia), WP (Greater Poland), SL (Silesia), PM (Pomerania), LD (Łódź), PK (Lesser Poland), ZP (West Pomerania), LB (Lubusz), KP (Kujawy-Pomerania), OP (Opole), PD (Podlaskie), SW (Świętokrzyskie), WM (Warmia-Masuria), PK (Podkarpackie), LU (Lubelskie).

In the frame of passive surveillance, a total of 115 body fluid samples from bats originating from 14 out of 16 Polish voievodeships were tested in the BioPro Rabies ELISA Ab kit (Figure 1). The largest number of samples originated from Silesia (SL) followed by Warmian-Mazurian (WM), Mazowian (MA), and Lower-Silesian (DS) (*n* = 10–16). Only a few samples were submitted from Lubusz (LB), Opole (OP), and Świętokrzyskie (SW) (Figure 1). While 46 (41.4%) and 65 (58.6%) were female and male, the sex of four bats could not be determined due to the decomposition stage of the carcasses and physical damages. Molecular classification based on cytochrome b genetic marker identified 89 bat specimens into 13 different bat species. The vast majority of bats comprised *Eptesicus serotinus* (*n* = 32; 35.96%), followed by *Nyctalus noctula* (*n* = 26; 29.2%) and *Vespertilio murinus* (*n* = 10; 11.2%), whereas only few other indigenous bat species were represented (Table 1). For 26 bats, genetic identification into species failed.

In total, 35 (30.4%; 95% confidence interval (CI): 22.8%–39.4%) out of 115 Polish bat samples tested positive in ELISA (Table 1). The percentage of positives in female was 44.68% (95% CI: 31.4%–58.76%), whereas, in males, it was 19.7% (95% CI: 11.89%–30.84%). Lyssavirus-specific antibodies were found in nine out of the 13 genetically identified bat species, mainly in *Nyctalus noctula* (*n* = 7), *Eptesicus serotinus* (*n* = 6), *Plecotus auritus* (*n* = 5), and *Myotis daubentonii* (*n* = 3), representing 25%, 21.4%, 17.8%, and 10.7% of all bats which tested seropositive in ELISA, respectively. For other bat species including *Myotis brandtii*, *Pipistrellus pipistrellus/Pipistrellus pygmaeus, Vespertillo murinus, Pipistrellus pipistrellus*, and *Barbastella barbastellus*, only single reactors were observed, while, for the remaining bats species, none of the samples available tested positive (Table 1). The two bats confirmed positive for EBLV-1 specific infection based on phylogenetic analysis of sequenced amplicons of heminested RT-PCR were negative in ELISA. 

**Figure 2:**
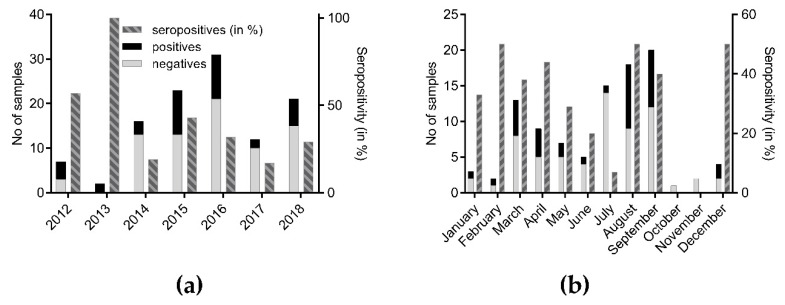
Total number of bat samples from Poland tested and seropositives (ELISA) thereof, stratified per year (**a**) and per month (**b**).

The majority of seropositive bats originated from Silesia (*n* = 10; 28.6%), Lower-Silesian (*n* = 7; 20%), Warmian-Mazurian (*n* = 5; 14.3%), and Mazowian (*n* = 4; 11.4%). The remaining 25.7% of seropositive bats were detected in West Pomeranian (ZP), Kyuavian Pomeranian (KP), Subcarpatian (PK), Opole (OP), Lublin (LU), and Great Poland (WP) voivodships (Figure 1). Seroprevalence (≥ 50%) was highest in bats from Lower Silesian, Opole, and Silesian mainly located in the southwestern parts of the country (Figure 1). The percentage of seropositive samples during the study in particular years ranged between 16.6% in 2017 and 100% in 2013 (Figure 2a). When stratified for months, the highest number of seropositive samples (>35%) was found in February–April, August–September, and December (Figure 2b).

### 3.2. Serological Testing of Eptesicus Bats (Eptesicus Fuscus and Eptesicus Serotinus) from Experimental Studies

**Figure 3:**
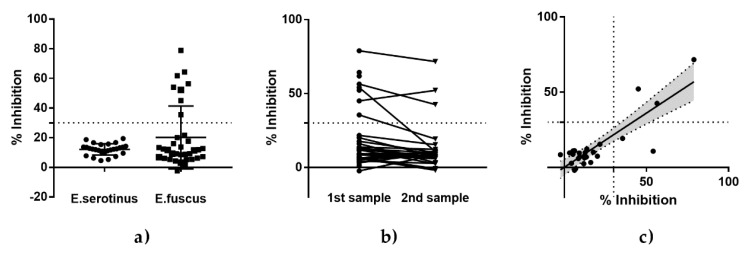
ELISA results (% inhibition) for sera from *Eptesicus serotinus* and *Eptesicus fuscus* taken prior to experimental studies. The threshold of 30% inhibition as a cut-off for seropositivity is indicated as a dashed line. (**a**) ELISA results for all sera tested stratified by species. For *Eptesicus fuscus,* serum was taken at two occasions prior to infection. The comparative results between sampling point one and two are presented on an individual level (**b**) and were analyzed by a linear regression model (**c**). The slope (solid line) and the 95% CI (gray zone) are indicated. The results for the second sampling can be calculated using the formula *Y* = 0.7205*X* + 0.07097, with a slope that is highly significant divergent from zero (*p* < 0,0001).

The serological status of *Eptesicus* bats with regard to lyssaviruses was estimated using both an immunoenzymatic method with the BioPro Rabies ELISA Ab Kit and a modified SNT with EBLV-1 as the test virus. All *Eptesicus serotinus* bats captured in Germany were negative for the presence of antibodies against lyssaviruses, both in ELISA (Figure 3a) and in RFFIT (Table S1, Supplementary Materials). In contrast, eight out of 35 American *Eptesicus fuscus* bats tested positive in ELISA (Figure 3a), although they had no EBLV-1-specific VNAs (<1:10). A comparison between two samplings revealed a slight decline in percentage inhibition values (Figure 3b), albeit with a high correlation (Figure 3c). 

## 4. Discussion

Although rabies in bats is the oldest known zoonotic disease associated with bats [2], our knowledge into various aspects of the disease and its association with bat biology is still limited. Partly, this is a result of unknown natural history of each bat species [42], their protected status [43], and the heterogeneous surveillance in Europe, terms of existing networks of bat biologists, the number of bat species submitted, and individual bats investigated [16]. Moreover, the discovery of novel lyssaviruses in European Natterer’s bats (BBLV, *Myotis nattereri*), Schreiber’s bent-winged bats (LLEBV, *Miniopterus schreibersii*), and Brandt’s bat (KBLV, *Myotis brandtii*) [5,9,12] highlights that additional bat species could serve as reservoirs for known or still unknown lyssaviruses in Europe. Therefore, the aim of this study was to enhance our knowledge on the epidemiology of the disease by analyzing the serological status of Polish bats against lyssaviruses, using available samples and a commercialized ELISA [34]. 

The seropositivity among Polish bats (30.4%) against lyssaviruses corroborates other European studies which demonstrated seroprevalence in insectivorous bat species (for a review, see Reference [16]). Specifically, the largest numbers of antibody positives were detected in *Nyctalus noctula* and *Eptesicus serotinus,* followed by *Plecotus auritus* and *Myotis daubentoni* (Table 1). While *Eptesicus serotinus* is the reservoir of EBLV-1, and seropositivity was detected in various countries for this bat species, the positive results in *Nyctalus noctula* are quite exceptional. In Germany, among more than 600 sera of this particular bat species tested, none rested positive in an SNT [21]. In the latter study, samples were taken from roosting animals, which may limit their contact to the rather anthropophilic *Eptesicus serotinus*. 

Daubenton’s bat (*Myotis daubentonii*) is the main reservoir of EBLV-2 [4], and EBLV-2 cases in bats were reported from several European countries [7]. Thus far, lyssaviruses reported from Poland were characterized as EBLV-1 or BBLV [8]. The detection of antibodies against lyssaviruses in two out of three Polish *Myotis daubentonii* could indicate the presence of EBLV-2 also in Poland, but an interspecies transmission of EBLV-1 from Serotine bats cannot be ruled out either. 

Generally, seasonality of seroprevalence in bats may result from differences in infectious status, behavioral ecology, and population dynamics among bat species [44]. In our study, the highest numbers of seropositive samples were found in August and September, as well as in March and April. The peak in seroprevalence in late summer seems to corroborate the assumption that the breeding period may favor virus transmission and subsequent infections, as demonstrated for *Eptesicus fuscus* [44]. Of note, significantly higher seroprevalences in this period were also demonstrated in France and Spain [25,45,46]. However, confounding effects cannot be excluded. For example, bat lyssavirus-specific antibodies were shown to persist up to four years in European bats, as exemplified for EBLV-1 [25]; thus, the antibodies detected in bats might not reflect recent exposure to lyssaviruses but might have been acquired much earlier. Additionally, the high degree of variation in seropositivity over time is partly influenced by a low sample size (Figure 2).

In our study, antibodies against lyssaviruses were detected in nine different bat species, some of which were not identified as reservoirs for lyssaviruses. These findings support hypotheses on (i) inter-species transmission of lyssaviruses from known reservoirs, (ii) an expansion of the reservoir range for one specific lyssavirus, or (iii) the potential presence of other novel lyssaviruses of Phylogroup I. Of note, our results should be carefully interpreted, and both the variable number of submissions and the limited dataset (Table, 1, Figure 2) prevent formal statistical analyses and drawing of general conclusions on the infection processes in insectivorous bats from Poland. 

The second objective of this study was to determine whether a commercially available BioPro ELISA Rabies Ab kit is suitable for bat samples. The seroprevalence in this study assessed with the ELISA is in the range of results from other European studies conducted with mFAVN or RFFIT, thus indicating the principle applicability of this approach for the detection of lyssavirus antibodies in bat samples in the frame of passive rabies surveillance and serological surveys (for a review, see Reference [16]). An unspecific binding of serum components that would lead to false positive results in the ELISA cannot be excluded; however, such a scenario was not reported during the broad usage of this ELISA in foxes or raccoon dogs [39]. This was corroborated by analyzing *Eptesicus serotinus* and *Eptesicus fuscus* sera from experimental studies. While all sera from *Eptesicus serotinus* that tested negative for EBLV-1-specific VNAs also tested negative in the ELISA (Figure 3a), eight sera from *Eptesicus fuscus* negative for EBLV-1 VNAs demonstrated a high concordance of results for subsequent samplings (Figure 3b,c). The fact that the ELISA-positive sera were SNT-negative is not surprising, as (i) the animals destined for infection studies with EBLV-1 were tested for homologous VNAs of the challenge virus and not for RABV-specific VNAs at the time [19], (ii) there is not necessarily a correlation between VNAs and binding antibodies [47], and (iii) cross-reactivity between EBLV-1 and RABV is less pronounced compared to other lyssaviruses [48].

The modified ELISA protocol as suggested by the manufacturer uses a smaller quantity of the sample (10 µL/sample as opposed to 50 µL/sample), which could allow for including small bat species into active bat rabies surveillance. Evidently, the higher dilution of samples results in a lower PB, as demonstrated by comparative analyses of ferrets experimentally infected with bat lyssaviruses (Figure S1a, Supplementary Materials). Even with a reduced cut-off for positivity of 30% PB, the sensitivity was reduced compared to the standard approach using a modified RFFIT. Notably, there was a low test agreement between the modified RFFIT and the ELISA, and only sera with a higher titer tended to test positive in the ELISA (Figure S1b,c, Supplementary Materials).

Obviously, a stringent validation, as was done in the development of the ELISA to be used for foxes and raccoon dogs for post-vaccination monitoring [39], is lacking for bats. Against the background of the genetic diversity of bats, the protected status of European bats, and the need for adaptation of cut-offs per species [47], this is rather illusionary. A balanced use of serial bleedings from an individual animal may represent an alternative to increase the robustness of sensitivity estimates of diagnostic tests, especially when sample availability is restricted [49]; however, in this case, it would require experimental vaccination or infection studies with different bat lyssaviruses.

Nonetheless, while negative results, therefore, do not exclude the presence of antibodies below the threshold of positivity, ELISA-positive sera appear to be an indicator for the presence of lyssavirus-specific antibodies.

Another advantage of the BioPro Rabies ELISA Ab kit is that it allows for the detection of antibodies in different matrices such as serum, plasma, or body fluids, and that it is not as sensitive to the quality of the samples as cell-based SNTs [34,50]. The BioPro ELISA Rabies Ab kit was specifically developed for the detection of antibodies against the RABV. However, because of cross-reactivity [51], ELISA may allow for the detection of antibodies against all lyssaviruses within phylogroup I. Further investigations and validations are necessary to standardize this method for detection of antibodies against RABV and non-RABV Lyssaviruses within phylogroup I in bat reservoirs.

Since RABV is the only lyssavirus present in the Americas [4], the approach from this study using an ELISA could ideally be used there, in the frame of serosurveillance in animals, particularly bats, thus providing highly reliable data. In the case of European and American bats, however, further studies are needed to verify the results obtained.

## Figures and Tables

**Table 1:** Results of the serological survey of Polish bats (2012–2018) on lyssavirus-specific antibodies using the BioPro Rabies ELISA Ab kit. CI—confidence interval.

Species	Females		Males		Total	
	N_pos_/N_total_	Positives % (95% CI)	N_pos_/N_total_	Positives % (95% CI)	N_pos_/N_total_	Positives % (95% CI)
*Eptesicus serotinus*	2/7	28.57% (5.08–64.11)	4/24	16.67% (6.68–35.85)	6/31	19.35% (9.19–36.28)
*Nyctalus noctula*	5/13	38.46% (17.71–64.48)	2/13	15.38% (2.73–42.23)	7/26	26.92%(13.7–46.08)
*Plecotus auritus*	2/2	100% (17.77–100)	3/4	75% (30.06–98.72)	5/6	83.33% (43.65–99.15)
*Hypsugo savii*	0		0/1	0% (0–94.81)	0/1	0% (0–94.81)
*Myotis dasycneme*	0/1	0% (0–94.81)	0		0/1	0% (0–94.81)
*Vespertillo murinus*	1/6	16.67% (0.85–56.35)	0/3	0% (0–56.15)	1/9	11.11% (0.57–43.5)
*Pipistrellus pipistrellus*	1/1	100% (5.13–100)	0/1	0% (0–94.81)	1/2	50% (2.56–97.44)
*P. pipistrellus/P. pygmaeus*	1/1	100% (5.13–100)	0		1/1	100% (5.13–100)
*Myotis daubentonii*	1/2	50% (2.56–97.44)	2/2	100% (17.77–100)	3/4	75% (30.06–98.72)
*Myotis brandtii*	1/1	100% (5.13–100)	1/1	100% (5.13–100)	2/2	2 (100)
*Barbastella barbastellus*	1/1	100% (5.13–100)	0		1/1	100% (5.13–100)
*Plecotus austriacus*	0/1	0% (0–94.81)	0		0/1	0% (0–94.81)
*Myotis nattereri*	0/1	0% (0–94.81)	0		0/1	0% (0–94.81)
n/a	5/10	50% (23.66–76.34)	1/17	5.88% (0.3–26.98)	6/27	22.22% (10.61–40.76)

## Supplementary Materials

The following are available online at https://www.mdpi.com/1999-4915/12/3/271/s1: Figure S1: Comparison of test results for sera from ferrets experimentally infected with EBLV-1. Table S1: All Eptesicus serotinus bats captured in Germany were negative for the presence of antibodies against lyssaviruses in RFFIT.
