# Supplementary material for: Serological Survey of Lyssaviruses in Polish Bats in the Frame of Passive Rabies Surveillance Using an Enzyme-Linked Immunosorbent Assay
Source: Viruses. 2020 Feb 28;12(3):271. doi: 10.3390/v12030271 (PMC7150987; doi:10.3390/v12030271)
Supplement: Supplementary file 1 [file viruses-12-00271-s001.zip › Supplementary Table 1.pdf]

FLI-ID N°.	Species	origin	Original-date	Original-Nr	RFFIT Titer	ProBIO ELISA			Animal trial N°	Reference
						OD-values	%-inhibition	ELISA		
12481	Eptesicus fuscus	USA	10.08.2005	66314	<1:10	0,5244	56,391	POS	745/2005	Franka, R., et al. (2008). "Susceptibility of North American big brown bats (Eptesicus fuscus) to infection with European bat lyssavirus type 1." Journal of General Virology 89: 1998-2010.
12482				66788	<1:10	1,0477	5,2704	NEG	745/2005	
12483				62859	<1:10	1,0388	6,1398	NEG	745/2005	
12484				60286	<1:10	1,1262	-2,3983	NEG	745/2005	
12485				60161	<1:10	1,0873	1,4018	NEG	745/2005	
12486				61682	<1:10	0,9762	12,255	NEG	745/2005	
12489				62076	<1:10	1,0472	5,3192	NEG	745/2005	
12490				62293	<1:10	0,4687	61,833	POS	745/2005	
12491				61131	<1:10	1,0485	5,1922	NEG	745/2005	
12492				67034	<1:10	1,0581	4,2544	NEG	745/2005	
12494				61400	<1:10	0,6412	44,981	POS	745/2005	
12495				58563	<1:10	0,9377	16,016	NEG	745/2005	
12498				59027	<1:10	0,9616	13,681	NEG	745/2005	
12499				62124	<1:10	0,9815	11,737	NEG	745/2005	
12500				60803	<1:10	0,5676	52,171	POS	745/2005	
12501				66944	<1:10	1,0382	6,1984	NEG	745/2005	
12502				60947	<1:10	0,9722	12,646	NEG	745/2005	
12503				60429	<1:10	1,0283	7,1655	NEG	745/2005	
12504				59501	<1:10	1,0083	9,1193	NEG	745/2005	
12505				63890	<1:10	0,7388	35,447	NEG	745/2005	
12506				59699	<1:10	1,0147	8,4941	NEG	745/2005	
12507				61399	<1:10	1,0315	6,8529	NEG	745/2005	
12508				61404	<1:10	0,4437	64,275	POS	745/2005	
12509				57612	<1:10	1,0094	9,0119	NEG	745/2005	
12510				65219	<1:10	0,9855	11,347	NEG	745/2005	
12512				61808	<1:10	0,8801	21,643	NEG	745/2005	
12513				67087	<1:10	0,5481	54,076	POS	745/2005	
12515				62057	<1:10	0,2945	78,85	POS	745/2005	
12532				58431	<1:10	0,8961	20,08	NEG	745/2005	
12533				61665	<1:10	0,9807	11,816	NEG	745/2005	
12534				66074	<1:10	0,9652	13,33	NEG	745/2005	
12535				65962	<1:10	1,0694	3,1505	NEG	745/2005	
12536				57030	<1:10	1,0071	9,2366	NEG	745/2005	
12537				60944	<1:10	0,921	17,648	NEG	745/2005	
12538				EF43 -	<1:10	1,0115	8,8067	NEG	745/2005	
13574			24.11.2005	61808	<1:10	1,0915	15,341	NEG	745/2005	
13575				67087	<1:10	1,1445	10,732	NEG	745/2005	
13576				62057	<1:10	0,4449	71,575	POS	745/2005	
13579				63890	<1:10	1,0465	19,255	NEG	745/2005	
13580				61131	<1:10	1,168	8,6881	NEG	745/2005	
13581				67034	<1:10	1,2366	2,7221	NEG	745/2005	
13583				58563	20/40	1,2305	3,2526	NEG	745/2005	
13584				59027	<1:10	1,1278	12,184	NEG	745/2005	
13585				61400	<1:10	0,6685	52,129	POS	745/2005	
13586				62859	<1:10	1,29	-1,922	NEG	745/2005	
13587				66074	<1:10	1,1896	6,8096	NEG	745/2005	
13589				57030	<1:10	1,1885	6,9052	NEG	745/2005	
13590				60944	<1:10	1,1516	10,114	NEG	745/2005	
13591				58431	<1:10	1,1824	7,4358	NEG	745/2005	
13592				65962	<1:10	1,1575	9,6013	NEG	745/2005	
13593				61665	n.a.	1,2402	2,409	NEG	745/2005	
13594				61399	<1:10	1,278	-0,87838	NEG	745/2005	
13595				60947	<1:10	1,155	9,8187	NEG	745/2005	
13596				59501	<1:10	1,1589	9,4795	NEG	745/2005	
13597				59699	<1:10	1,2015	5,7747	NEG	745/2005	
13598				60286	<1:10	1,1715	8,3837	NEG	745/2005	
13599				66944	<1:10	1,1411	11,028	NEG	745/2005	
13600				61682	<1:10	1,1942	6,4095	NEG	745/2005	
13601				66788	<1:10	1,1426	10,897	NEG	745/2005	
13602				66314	<1:10	0,7791	42,51	POS	745/2005	
16816	Eptesicus serotinus	Germany	16.08.2007	31923	n.a.	0,9309	16,681	NEG	1085/2007	Freuling, C., et al. (2009). "Experimental infection of serotine bats (Eptesicus serotinus) with European bat lyssavirus type 1a." Journal of General Virology 90(10): 2493-2502.
16818				29725	<1:10	0,9994	9,9888	NEG	1085/2007	
16819				33841	<1:10	0,9559	14,238	NEG	1085/2007	
16820				27896	n.a.	0,9841	11,483	NEG	1085/2007	
16821				29107	<1:10	0,9447	15,332	NEG	1085/2007	
16822				34550	<1:10	1,0041	9,5296	NEG	1085/2007	
16823				25599	n.a.	0,9709	12,773	NEG	1085/2007	
16825				32475	<1:10	1,0549	4,567	NEG	1085/2007	
16826				32631	<1:10	0,982	11,689	NEG	1085/2007	
16828				34620	n.a.	0,9756	12,314	NEG	1085/2007	
16829				34526	<1:10	1,0265	7,3414	NEG	1085/2007	
18186				29579	n.a.	1,022	7,781	NEG	1085/2007	
18187				31088	<1:10	0,9626	13,584	NEG	1085/2007	
18188				31263	<1:10	1,0478	5,2606	NEG	1085/2007	
18189				32990	<1:10	0,903	19,406	NEG	1085/2007	
18190				29033	<1:10	0,964	13,447	NEG	1085/2007	
18191				29614	<1:10	0,9313	16,641	NEG	1085/2007	
18192				32373	<1:10	0,9771	12,167	NEG	1085/2007	
18194				30896	<1:10	0,9105	18,673	NEG	1085/2007	
18195				31215	<1:10	0,9614	13,701	NEG	1085/2007	
18196				32361	<1:10	1,0383	6,1886	NEG	1085/2007	
18197				29460	<1:10	0,9869	11,21	NEG	1085/2007	
18198				34392	<1:10	0,9406	15,733	NEG	1085/2007	
18199				33342	<1:10	0,9674	13,115	NEG	1085/2007	
18200				30892	n.a.	0,9963	10,292	NEG	1085/2007	
18201				27030	<1:10	0,9796	11,923	NEG	1085/2007	
18202				31638	<1:10	0,9742	12,451	NEG	1085/2007	
