# Supplementary figures and images for: Serological Survey of Lyssaviruses in Polish Bats in the Frame of Passive Rabies Surveillance Using an Enzyme-Linked Immunosorbent Assay
Source: Viruses. 2020 Feb 28;12(3):271. doi: 10.3390/v12030271 (PMC7150987; doi:10.3390/v12030271)

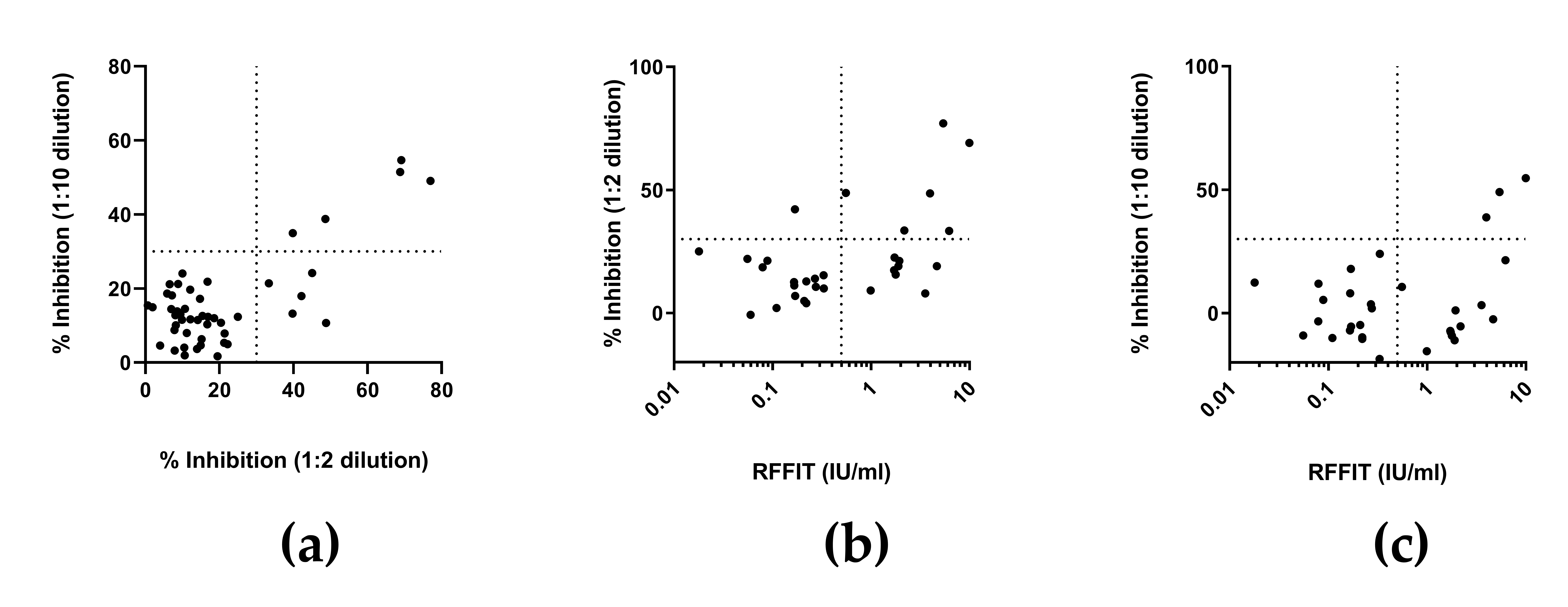

Supplement: Supplementary file 1 [file viruses-12-00271-s001.zip › viruses-684380-supplementary.png]
